# Preferential and enhanced adsorption of methyl green on different greenly synthesized magnetite nanoparticles: investigation of the influence of the mediating plant extract’s acidity

**DOI:** 10.1039/d2ra01085b

**Published:** 2022-05-13

**Authors:** Kaouthar Ahmouda, Boubaker Benhaoua

**Affiliations:** Department of Process Engineering and Petrochemistry, Faculty of Technology, University of El Oued El Oued 39000 Algeria ahmouda-kaouthar@univ-eloued.dz; Renewable Energy in Arid Zones Research Unit, University of El Oued El Oued 39000 Algeria; Department of Physics, Faculty of Exact Sciences, University of El Oued El Oued 39000 Algeria benhaouab@yahoo.fr

## Abstract

Four magnetite nanoparticle (NP) samples have been greenly synthesized using four aqueous plant extracts, which are *Artemisia herba-alba* (L), *Rosmarinus officinalis* (L), *Matricaria pubescens* (L), and *Juniperus phoenicia* (L). The pH of these extracts are acidic (5.25, 5.05, 4.63, and 3.69, respectively). The synthesized samples were characterized by XRD, SEM, ATR-FTIR, and UV-Vis. This work aimed to study the preferential and enhanced adsorption of methyl green (MG) on the four greenly synthesized Fe_3_O_4_ surfaces by coupling three processes: MG adsorption in ambient dark conditions as the first process, followed by the thermocatalysis of the MG/Fe_3_O_4_ residual solution in the second process, and finally photocatalysis by the UV irradiation of MG/Fe_3_O_4_ residual solution after carrying out thermocatalysis. The novelty of this study lies in highlighting the influence of the mediating plant extract’s acidity on the magnetite NPs’ physicochemical characteristics, which impact the preferential and enhanced MG adsorption. The studied physicochemical characteristics are the functional hydroxyl group density on the magnetite surface, grain size, and band gap energy. It was found that the plant extract’s acidity has a clear effect on the studied physicochemical properties. The analysis of the FTIR spectra showed that the hydroxyl group densities differ on the four magnetite samples. Furthermore, the calculated grain sizes of the magnetite samples based on XRD spectra data vary from 29.27 to 41.49 nm. The analysis of the UV-Vis spectra of the four magnetite samples showed that the estimated direct band gap energies vary from 2.87 to 2.97 eV. The obtained results showed that the decrease of the mediating plant extract’s acidity leads to an increase in the hydroxyl group density on magnetite surfaces, which resulted in an increase in the MG adsorption capacities and yields in the first process of adsorption. Thus, MG adsorption was more preferred on greenly synthesized magnetite surfaces mediated by plant extracts with low acidity (*Artemisia herba-alba* (L) and *Rosmarinus officinalis* (L)). Furthermore, the increase of the plant extract’s acidity leads to a decrease in the particle size and an increase in the band gap energy and, therefore, to the decrease of the electron/hole pair recombination speed upon electron excitation. So, magnetite greenly synthesized from a more acidic mediating plant extract showed higher thermo- and photocatalytic activities for MG adsorption (*Juniperus phoenicia* (L) and *Matricaria pubescens* (L)). However, under photocatalysis, the enhancement is even more significant compared to thermocatalysis.

## Abstract 2

Magnetite NP samples synthesized from less acidic plant extracts have more dense active sites and prefer adsorbing more MG. The increase of plant extract acidity leads to the increase of the thermo- and photocatalytic activities of Fe_3_O_4_ NPs.

## Graphical abstract

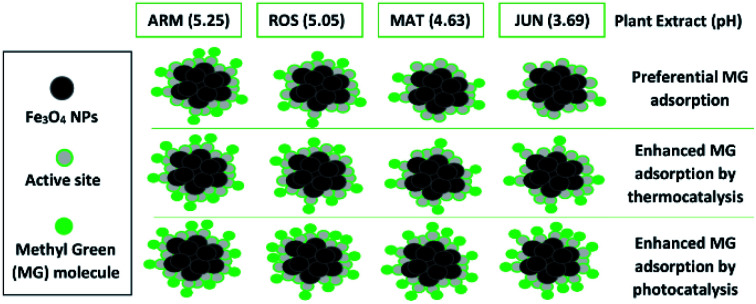

## 1 Introduction

Nanomaterials are widely used in the purification of aqueous media.^1–4^ They allow a rapid thermodynamic equilibrium between adsorbent and adsorbate during the adsorption process and the selective removal of pollutants.^5–7^ Adsorption has been extensively studied as a cost-effective process for removing a wide variety of pollutants from aqueous solutions, such as dyes.^8–10^ The adsorption ability of iron oxide NPs arises from the intervention of hydroxyl groups during pollutant dissociation.^11^

Surface hydroxyl groups, with amphoteric properties, are the functional groups of iron oxide surfaces and they are the chemically reactive entities that behave as the active sites in the adsorption process. These hydroxyl groups may be singly, doubly, and triply coordinated to Fe atoms, with different reactivities. The overall density of these groups depends on both the crystal structure and the extent of the development of the different crystal faces.^12^

Photo- and thermocatalysts absorb photons/phonons with an energy equal to or more than the band gap energy between the valence band (VB) and conduction band (CB) of the photo- or thermocatalyst. Photon/phonon absorption causes charge separation by exciting electrons from the VB to the CB, followed by the generation of positive holes in the VB.^13,14^ These positive holes oxidize adsorbed H_2_O molecules and produce hydroxyl radicals (OH˙). Whereas excited electrons reduce the adsorbed O_2_ in the CB and produce hydroxyl radicals (OH˙). These OH˙ radicals attack the organic groups of the pollutant and undergo various reactions to convert the organic pollutants into non-toxic and non-hazardous forms or completely degrade them into CO_2_ and H_2_O.^14,15^ The photo- and thermogenerated electron/hole pairs exhibit a strong tendency to recombine. Recombination lifetime speed is an important factor that influences the photo- and thermocatalysis efficiency. If the recombination of photo- and thermogenerated charges is slow, then the photo- and thermocatalytic degradation of pollutants is more efficient.^16^

Several works have studied the thermo- and photocatalysis of dye adsorption on nanomaterials, and they reported the high-efficiency thermo- and photocatalytic activities of nanomaterials. Wu *et al.*^17^ studied the thermocatalysis of methylene blue adsorption on magnetite Fe_3_O_4_@C NPs. They found that an increase of temperature leads to an increase of methylene blue thermodegradation, which indicates the high thermocatalytic activity of the studied nanomaterial. Other authors^18^ studied the thermocatalysis of N719 dye on anatase TiO_2_ nanosheets with dominant (001) facets and TiO_2_ NPs with dominant (101) facets. They found that an increase of temperature leads to an increase of N719 dye thermodegradation on both studied nano-adsorbents due to the thermocatalytic activity of TiO_2_ NPs. Farghali *et al.*^19^ studied the thermocatalysis of methylene blue on multi-walled carbon nanotubes decorated with CoFe_2_O_4_ NPs by increasing the temperature. They reported that this nanocomposite showed efficient thermocatalytic activity.

Furthermore, Ge *et al.*^20^ studied the photocatalysis of methylene blue and methyl orange adsorption on iron oxide anchored to single-wall carbon nanotubes by UV irradiation. They reported that the studied adsorbent showed efficient photocatalytic activity. Elhadj *et al.*^21^ studied the photocatalysis of Basic Red 46 dye adsorption over ZnO NPs under solar irradiation. They reported that ZnO NPs exhibit high photocatalytic activity. Moreover, Kumar *et al.*^22^ studied the photodegradation of methylene blue (MB), Congo red (CR), and methylene orange (MO) under sunlight irradiation in the presence of greenly synthesized magnetite mediated by Andean blackberry leaf extract. They reported that the presence of those magnetite NPs accelerated the photodegradation of the three dyes due to their high photocatalytic activity. Sirdeshpande *et al.*^23^ studied the photodegradation of malachite green under sunlight irradiation in the presence of greenly synthesized magnetite using leaf extract of *Calliandra haematocephala*. They reported that the presence of those magnetite NPs increased the photodegradation of malachite green. Other authors^24^ compared the photocatalytic activity of several composites of titanium dioxide containing magnetite NPs with different morphologies and structures in the photodegradation of Rhodamine B by UV irradiation. They reported that the highest dye photodegradation was observed when both spherical and rod-shaped composite structures based on titanium dioxide containing 1 wt% of magnetite NPs were used as a photocatalyst. Jassal *et al.*^25^ studied the thermo- and photodegradation of malachite green (MG) and Eriochrome Black T (EBT) dyes on greenly synthesized potassium zinc hexacyanoferrate nanocubes. They found that this adsorbent acted as a photocatalyst, not a thermocatalyst.

Several parameters can impact photo- and thermocatalysis processes, such as solution pH, adsorbent concentration, dye concentration, solution ionic strength, temperature,^25–28^ dye structure properties,^29,30^ adsorbent particle size,^31^ gap energy, recombination lifetime of the electron/hole pairs,^32,33^ adsorbent type,^34,35^ light source and time of light exposure.^34^ Ullah *et al.*^32^ reported that a Mn^2+^ dopant in the ZnO NPs decreased the recombination of the electron/hole pairs, which enhanced the photocatalytic activity efficiency for the removal of dyes. Rafaie *et al.*^33^ studied the photocatalytic properties of ZnO NPs microstructures decorated with Ag NPs for the degradation of methylene blue under UV irradiation. They reported that the Ag NPs played the role of electron sinks and trapped the photogenerated electrons, which increased the electron/hole pair lifetime. As a result, the ZnO–Ag nanostructure exhibited higher photocatalytic activity for the degradation of MB dye.

Saha *et al.*^5^ studied the preferential adsorption of seven different dyes on magnetite NPs. They reported that the magnetite surface preferred adsorbing dyes containing higher OH content. Xiao *et al.*^36^ studied the preferential adsorption of different cationic and anionic dyes on iron NPs. They reported that iron NPs preferred removing cationic dyes over anionic dyes. Madrakian *et al.*^37^ studied the preferential adsorption of seven cationic and anionic dyes on magnetite-coated waste tea. They reported that the adsorption capacities of these NP adsorbents for the adsorption of cationic dyes were more increased compared to those for anionic dyes.

Several factors can influence the adsorption, such as the solution pH,^12^ solution ionic strength,^38^ dye concentration,^39^ magnetite NP concentration,^5^ and hydroxyl group density on the adsorbent surface.^40^ The impact of changing plants on greenly synthesized metal oxide NPs’ reactivity in dye adsorption has been studied in several works. Huang *et al.*^41^ studied the effect of three different tea extracts (green, oolong, and black teas) on the properties of iron oxide NP surfaces and their reactivities in the removal of methyl green from aqueous solutions. They reported that the plant extract has an effect on the reactivity of the iron oxide NP surfaces, with 81.2%, 75.6%, and 67.1% of methyl green dye being removed by iron oxide NPs synthesized using the extracts of green, oolong, and black teas, respectively. Likewise, Xiao *et al.*^36^ studied the removal of six cationic and anionic dyes. They reported that iron NPs greenly synthesized with tea extract showed preferential adsorption of cationic dyes from an aqueous solution. Other authors^42^ synthesized metal oxide NPs using the extracts of flowers, bark, and the leaf of *Tecoma stans* in order to use them in the removal of Congo red (CR) and crystal violet (CV) dyes. They reported that the adsorbent derived from flower extract gave better dye adsorption efficiency than those derived from other extracts. Furthermore, Islam *et al.*^43^ synthesized magnetite NPs using six plant extracts in order to use them in the removal of methyl orange (MO) and crystal violet (CV) dyes. They reported that the plant extract had an effect on the magnetite NPs’ surface reactivity in the adsorption, where magnetite NPs synthesized using tea extract showed the highest performance (MO 92.34%, CV 96.1%).

In this paper, the preferential and enhanced adsorption of MG on four greenly synthesized Fe_3_O_4_ NP surfaces has been studied by coupling three processes. The preferential adsorption of MG on the four magnetite surfaces in ambient dark conditions is the first process, followed by the adsorption enhancement by the thermocatalysis of MG/Fe_3_O_4_ residual solutions in dark conditions at the second process, and finally the adsorption enhancement by photocatalysis under UV irradiation (365 nm) in ambient conditions of the MG/Fe_3_O_4_ residual solutions after thermocatalysis. The focus of this study is the investigation of the influence of the mediating plant extract’s acidity on the greenly synthesized magnetite NPs’ physicochemical characteristics, which impact the preferential and enhanced MG adsorption. The studied physicochemical characteristics are the functional hydroxyl group density on the magnetite surfaces, grain size, and band gap energy. The mediating plants in the green synthesis are *Artemisia herba-alba* (L), *Matricaria pubescens* (L), *Juniperus phoenicia* (L), and *Rosmarinus officinalis* (L), and synthesized Fe_3_O_4_ samples from their extracts are respectively denoted in this paper as ARM-Fe_3_O_4_, MAT-Fe_3_O_4_, JUN-Fe_3_O_4_ and ROS-Fe_3_O_4_. The Fe_3_O_4_ NP samples were characterized by XRD, SEM, FTIR-ATR, and UV-Vis techniques. In preferential MG adsorption, the pseudo-first-order and pseudo-second-order kinetics of the adsorption, as well as the intra-particle diffusion mechanism, have been analyzed. Under thermocatalysis, the activated thermodynamic parameters of free energy (Δ*G*^0^), entropy (Δ*S*^0^), enthalpy (Δ*H*^0^), and activation energy (*E*_a_) have been analyzed. Under photocatalysis, the pseudo-first-order kinetics have also been analyzed.

The remainder of this paper is organized as follows. The next section gives a description of the materials used and methods followed during experiments. In Section 3, the obtained results are presented and discussed by analyzing the XRD, SEM, FTIR-ATR, and UV-Vis data. Section 3.7 is then devoted to presenting the effect of the plant extract’s acidity on the physicochemical properties of greenly synthesized Fe_3_O_4_ in the preferential and enhanced methyl green adsorption. The last section presents the conclusions.

## 2 Materials and methods

This section focuses on listing the materials needed and apparatuses used. It also provides details of the methods utilized to perform the adsorption experiments and characterization of the iron oxide NPs.

### 2.1 Materials

#### 2.1.1 Chemicals

**Fig. 1:**
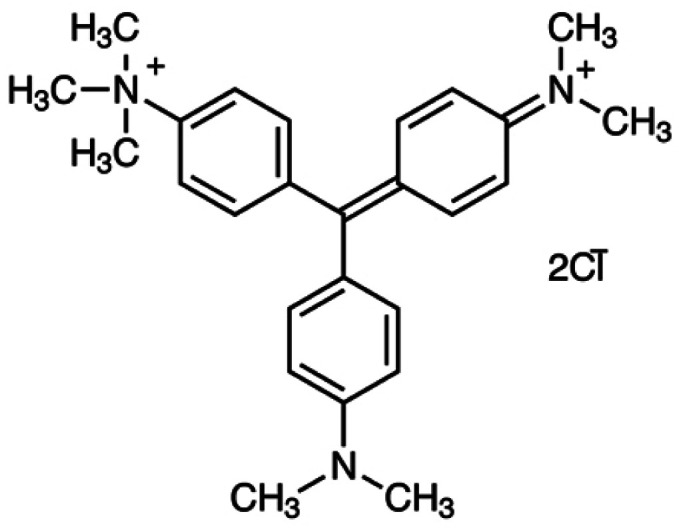
Methyl green structure.

Methyl green dye, NaCl salt, and HCl acid were purchased from Sigma-Aldrich. JUN-Fe_3_O_4_, ROS-Fe_3_O_4_, MAT-Fe_3_O_4_ and ARM-Fe_3_O_4_ NP powders were greenly synthesised using an iron salt (FeCl_3_·6H_2_O) (purchased from Biochem Chemopharma Co, Canada) as the precursor and *Artemisia herba-alba* (L) (Asteraceae family), *Matricaria pubescens* (L) (Asteraceae family), *Juniperus phoenicia* (L) (Cupressaceae family), *Rosmarinus officinalis* (L) (Lamiaceae family) plants as reducing agents. Fe_3_O_4_ samples were obtained after 4 months of storage of the synthesized iron oxides in ambient conditions. The freshly synthesized samples were both wüstite and magnetite.^67^ The chemical structure of MG is presented in Fig. 1.

#### 2.1.2 Apparatuses

An XPERT-PRO X-ray diffractometer (RigakuMiniflex 600) with conditions of 30 keV and 30 mA for X-ray generation and the Kα radiation of copper *λ* = 1.54056 Å was used. Fourier transform infrared spectroscopy (FTIR-ATR): Shimadzu IR-Infinity. UV-Vis spectroscopy: Shimadzu UV-Vis spectrophotometer apparatus Model 1800 operating in the range of 200–900 nm. Instantaneous global UV (direct plus diffuse) was measured with a UV radiometer *λ*_max_ = 365 nm VL.215. L (Ecosystem-Environmental Services, France).

### 2.2 Methods

In this section, the methods used for solution preparation are described. The protocol used in the adsorption experiments of iron oxide NPs and characterization techniques are described as well.

#### 2.2.1 Batch adsorption experiments of MG on magnetite surfaces in ambient dark conditions

In the first step, the prepared standard aqueous solutions of MG dye were diluted several times as required. In the second step, 0.0015 g of JUN-Fe_3_O_4_, ROS-Fe_3_O_4_, MAT-Fe_3_O_4_ and ARM-Fe_3_O_4_ NP powders were added to a volume of 4 ml of the aqueous solution of the dye. The dye solution concentration was 0.0111 mg ml^−1^. The ionic strength for all adsorption experiments was kept at 0.1 M by adding an appropriate amount of NaCl (0.023 g). A dilute solution of HCl was used to adjust the dye/Fe_3_O_4_ solution pH to 4. This protocol was used to prepare, in total, 44 experiment sets (11 for each magnetite sample). In addition, 4 control experiment sets (without NPs) were also prepared.

All experiment sets were sonicated in an ultrasonic bath for 15 minutes and they were then stirred continually for 60 minutes until a steady state was reached. All adsorption experiments were carried out in ambient dark conditions in batch mode, and they were performed in triplicate for data consistency.

Kinetic experiments were performed by withdrawing samples of the MG/Fe_3_O_4_ solutions at regular time intervals to obtain, after centrifugation, adequate aliquots for the purpose of quantifying the residual dye concentrations and the adsorbed amounts. The concentrations of the aqueous solutions of the residual dye were quantified using a UV-Vis spectrophotometer at an absorbance maxima of MG *λ*_max_ = 249 nm. Furthermore, the adsorbed amounts of MG molecules were calculated from the calibration curve for all adsorption experiments (*Y* = 42.049*X* − 0.2885, *R*^2^ = 0.996). In order to obtain the adsorption capacity *q*_eI_ (mg g^−1^) and the amount of MG cations adsorbed per unit mass (*q*_tI_ in (mg g^−1^)) of magnetite NPs at the equilibrium contact time in the first process of MG adsorption, the following equations were used:1
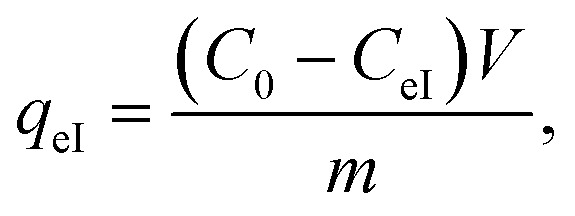
2
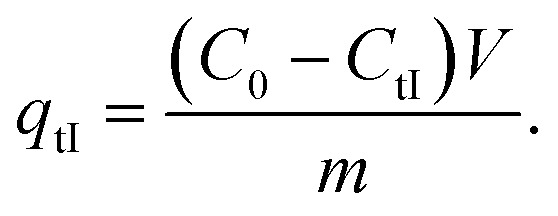


Adsorption yield was calculated using the following equation:3
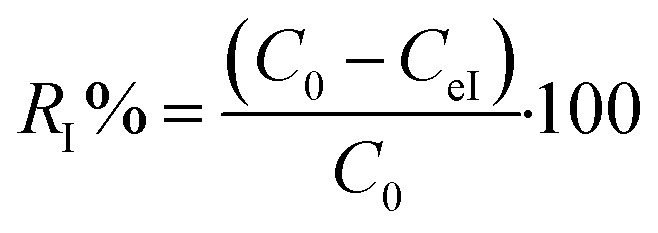
where *C*_0_, *C*_eI_, *C*_tI_, *V* and *m* are, respectively, the initial dye concentration without any treatment (mg ml^−1^), residual dye concentration in the liquid phase at steady state after the first process of MG adsorption (mg ml^−1^), residual dye concentration in the liquid phase at steady state after the accomplishment of the first process of MG adsorption (mg ml^−1^) at time *t*, the volume of dye solution (ml), and the amount of magnetite NPs (g).

#### 2.2.2 Pseudo-first-order and pseudo-second-order kinetics

The pseudo-first-order (PFO) of Lagergren^44^ and pseudo-second-order of Ho and Mckay^45^ kinetic models were selected to test the adsorption dynamics in this study due to their good applicability in most studies.^46,47^ The Lagergren kinetic model assumes that the rate of the occupation of adsorption sites is proportional to the number of unoccupied sites.^48^ Lagergren’s model (eqn (5)) is suitable for only the initial 20 to 30 minutes of the adsorption action and not for the whole range of contact times.^45^ It is generally represented by the following equation:4
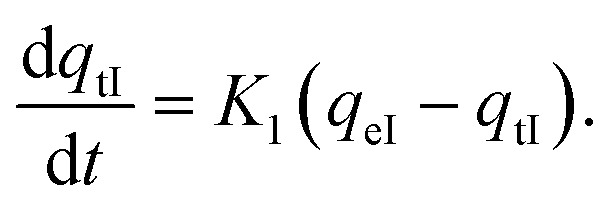


After integration by the conditions *q*_tI_ = 0 at *t* = 0 and *q*_*t*_ = *q*_tI_ at *t* = *t*, then eqn (4) becomes:

5 ln(*q*_eI_−*q*_tI_) = ln*q*_eI_−*K*_1_,

where *K*_1_, *q*_tI_ and *q*_eI_ are, respectively, the pseudo-first-order kinetic constant (mn^−1^), adsorbed dye quantity at time *t* (mg g^−1^) and adsorbed dye quantity at thermodynamic equilibrium in ambient dark conditions (mg g^−1^).

If the active surface of the adsorbent is regarded as invariable, the reaction could be treated as pseudo-first-order. However, once the active sites have been saturated, the transfer at the pollutant/adsorbent particle interface may be limited by mass transfer.^49^

The pseudo-second-order (PSO) model (eqn (6)) is proposed by Ho and McKay.^45^ It is based on the adsorption capacity, expressed as follow:6
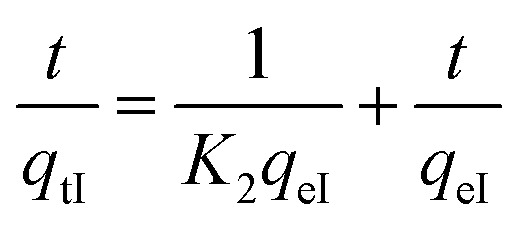
where *K*_2_, *q*_tI_ and *q*_eI_ are the pseudo-second-order kinetic constant (mg g^−1^ mn^−1^), adsorbed dye quantity at time *t* (mg g^−1^) and adsorbed dye quantity at thermodynamic equilibrium in the first process (mg g^−1^), respectively.

#### 2.2.3 Intra-particle diffusion kinetics

In order to gain insights into the adsorption mechanisms involved, a homogeneous particle diffusion model (HPDM), as shown in eqn (7), originally proposed by Boyd *et al.*,^50^ is used to describe the diffusive adsorption process. In this model, the rate-limiting step is usually described by either an intra-particle diffusion or a film diffusion mechanism.7
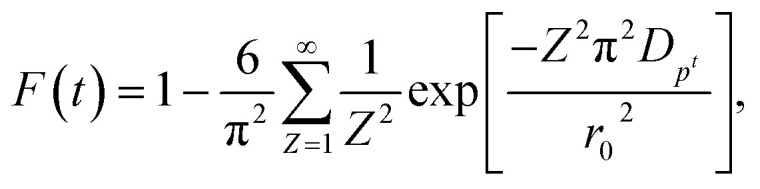
where *F*(*t*) is the fractional attainment at time *t*, *i.e.*, *F*(*t*) = *q*_tI_/*q*_eI_, *D*_p_ (m^2^ s^−1^) is the effective diffusion coefficient, *r*_o_ is the radius of Fe_3_O_4_ particles, which are assumed to be spherical, and *Z* is an integer. For 0 < *F*(*t*) < 1, a simplified equation can be obtained for the adsorption on spherical particles:8
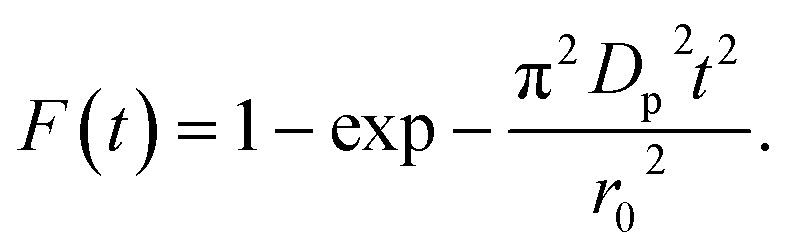


A further formula alteration gives the following:9
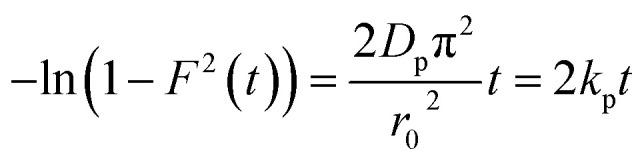
where *k*_p_ is the diffusion rate constant (1/*s*) and *k*_p_ = *D*_p_π^2^/*r*_0_^2^. Eqn (9) was used for the calculation of the effective intra-particle diffusivity (*D*_p_ (m^2^ s^−1^)) from the experimental data. In the first step, a graph of −ln(1 − *F*^2^(*t*)) *vs. t* was produced. The values of *k*_p_ of the MG/ARM-Fe_3_O_4_, MG/ROS-Fe_3_O_4_, MG/MAT-Fe_3_O_4_, and MG/JUN-Fe_3_O_4_ adsorption processes were obtained from the slopes of the fitted lines (plots of −ln(1 − *F*^2^) *vs.* time), and the values of the effective diffusion coefficients, *D*_p_ (m^2^ s^−1^), could then be obtained from *D*_p_ = *k*_p_π^2^/*r*_0_^2^.

Additionally, eqn (10) can be used when the rate of adsorption is controlled by liquid film diffusion.^51^10
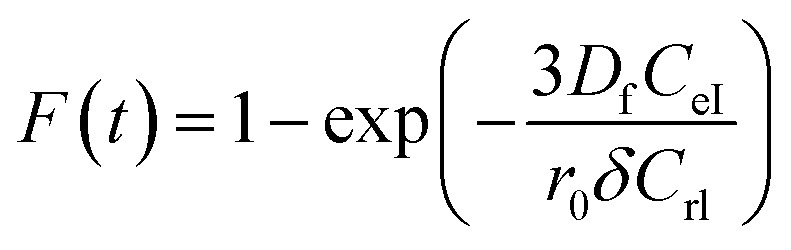
where *D*_f_ is the film diffusion coefficient (m^2^ s^−1^) in the liquid phase, and *C*_eI_ (mol l^−1^) and *C*_rI_ (mol l^−1^) are, respectively, the equilibrium concentrations of MG dye in the solution and solid phases. *δ* is the thickness of the liquid film, which was assumed to be 10^−5^ m according to Yu and Luo.^52^ A further formula alteration of eqn (10) gives the following equation:

11 −ln(1 − *F*) = *K*_f_*t*

where *k*_f_ is the diffusion rate constant (1/*s*).

The values of *k*_f_ = 3*D*_f_*C*_eI_/*r*_0_*δC*_rI_ of the MG/ARM-Fe_3_O_4_, MG/ROS-Fe_3_O_4_, MG/MAT-Fe_3_O_4_, and MG/JUN-Fe_3_O_4_ adsorption processes were obtained from the slopes of the fitted lines (plots of −ln(1 − *F*) *vs.* time), and the values of the effective diffusion coefficient, *D*_f_(*m*^2^ s^−1^), could then be obtained from *D*_f_ = *k*_f_*r*_0_*C*_rI_/3*C*_eI_.

The linearity test of Boyd plots (−ln(1 − *F*) and −ln(1 − *F*^2^) *versus* time plots) was employed to distinguish between the film diffusion and particle diffusion-controlled adsorption mechanisms. If the plot of −ln(1 − *F*) *versus* time is a straight line passing through the origin, then the adsorption rate is governed by the particle diffusion mechanism; otherwise, if −ln(1 − *F*^2^) *versus* time is a straight line passing through the origin, then the adsorption is governed by film diffusion.

#### 2.2.4 Batch thermocatalysis experiments of the magnetite samples

In order to study the thermocatalysis of JUN-Fe_3_O_4_, ROS-Fe_3_O_4_, MAT-Fe_3_O_4_ and ARM-Fe_3_O_4_ NPs under heat, all sets of experiments containing residual solutions after the first process of MG adsorption were sonicated in an ultrasonic bath for 15 min and then stirred continually for 20 minutes in dark conditions at different temperatures ranging from 303.15 to 318.15 K. The concentrations of residual MG dye in the liquid phase were quantified using a UV-Vis spectrophotometer at an absorbance maxima of MG *λ*_max_ = 249 nm. Furthermore, the adsorbed amounts of MG molecules were calculated from the calibration curve for all adsorption experiments (*Y* = 42.049*X* − 0.2885, *R*^2^ = 0.996). In order to obtain the adsorption capacity *q*_eII_ (mg g^−1^) of all magnetite samples after carrying out thermocatalysis in the second process of MG adsorption in dark conditions, the following equation was used:12
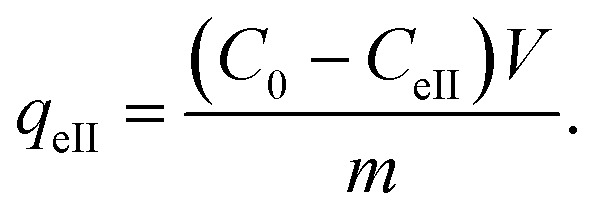


Adsorption yield was calculated using the following equation:13
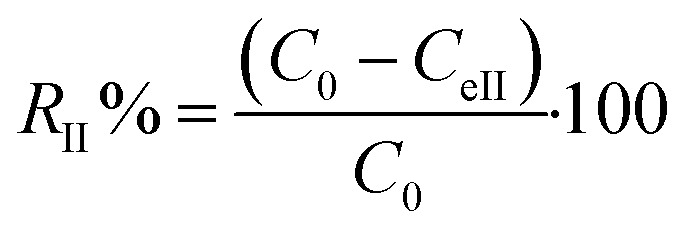
where *C*_0_, *C*_eII_, *V* and *m* are, respectively, the initial dye concentration without any treatment (mg ml^−1^), residual dye concentration in the liquid phase at steady state in the first process of MG adsorption (mg ml^−1^), the volume of dye solution (ml), and the amount of magnetite NPs (g).

The activated enthalpy (Δ*H*^0^) of MG adsorption on the magnetite NP surface was determined using the Arrhenius equation as follows:14
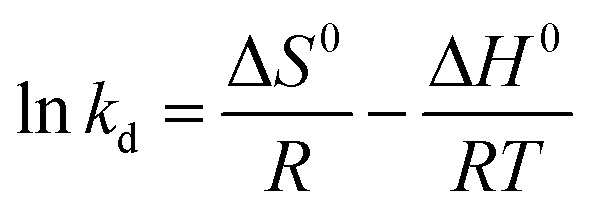
where *R* (1.987 cal mol^−1^ K^−1^) is the universal gas constant, *T* is the absolute solution temperature (K), and *K*_d_ is the distribution coefficient, which can be calculated as:15
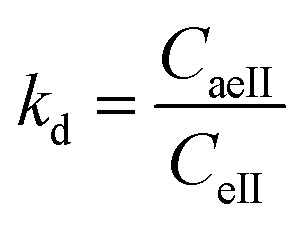
where *C*_aeII_ (mg ml^−1^) and *c*_eII_ (mg ml^−1^) are, respectively, the concentration of adsorbed dye on the solid and the dye residual concentration in the liquid phase after thermocatalysis in dark conditions.

The values of activated enthalpy (Δ*H*^0^) and entropy (Δ*S*^0^) were calculated from the slope and intercept of the plot of ln *K*_d_*versus* 1/*T*. Δ*G*^0^ was then calculated using the following equation:

16 Δ*G*^0^ = −*RT* ln *K*_d_.

The free energy change indicates the degree of the spontaneity of the adsorption process and the higher negative value reflects more energetically favorable adsorption. The activation energy (Δ*E*_a_) of MG adsorption on magnetite surface is determined using the following Arrhenius’s equation:17
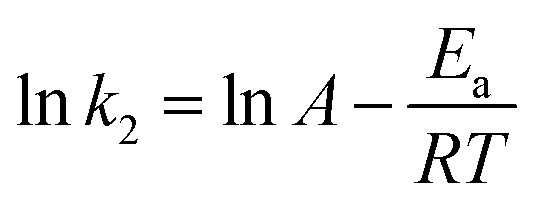
where *K*_2_ is the distribution coefficient which can be calculated by:18
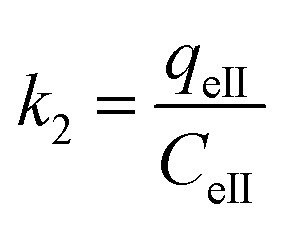
where *q*_eII_ (mg g^−1^) and *c*_eII_ (mg ml^−1^) are, respectively, the adsorption capacity of the dye on the solid and the dye residual concentration in the liquid phase after carrying out thermocatalysis in the second process of the MG adsorption in dark conditions.

#### 2.2.5 Batch photocatalysis experiments of magnetite NP samples in ambient conditions

In the third MG adsorption process, the study of the photocatalysis of JUN-Fe_3_O_4_, ROS-Fe_3_O_4_, MAT-Fe_3_O_4_ and ARM-Fe_3_O_4_ NPs under UV irradiation to degrade MG was conducted on all experimental sets containing residual solutions after carrying out thermocatalysis in dark conditions. All experiment sets were sonicated in an ultrasonic bath for 15 minutes and then they were stirred continuously and exposed to direct UV irradiation (365 nm) in ambient conditions for different times ranging from 60 to 240 minutes. The concentrations of the residual dye aqueous solutions were quantified using a UV-Vis spectrophotometer at an absorbance maxima of MG *λ*_max_ = 249 nm. Furthermore, the adsorbed amounts of MG molecules were calculated from the calibration curve for all adsorption experiments (*Y* = 42.049*X* − 0.2885, *R*^2^ = 0.996). In order to obtain the adsorption capacity *q*_eIII_ (mg g^−1^) by photocatalysis in ambient conditions under the UV irradiation of JUN-Fe_3_O_4_, ROS-Fe_3_O_4_, MAT-Fe_3_O_4_ and ARM-Fe_3_O_4_, the following equation was used:19
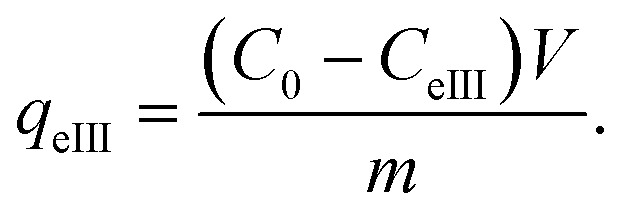


Adsorption yield was calculated using the following equation:20
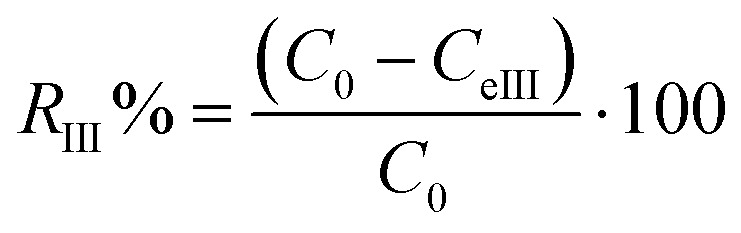
where *C*_0_, *C*_eIII_, V and *m* are, respectively, the initial dye concentration without any treatment (mg ml^−1^), the dye residual concentration in liquid phase after photocatalysis in the third process of MG adsorption in ambient conditions (mg ml^−1^) under UV irradiation, the volume of the dye solution (ml), and the amount of magnetite (g).

The degradation kinetics of MG using Fe_3_O_4_ NPs can be expressed as a pseudo-first-order (PFO) reaction as follows:

21 ln(*C*_0_/*C*_tIII_) = *k*_pd_·*t*

where *C*_0_, *C*_tIII_, and *k*_pd_ are, respectively, the initial concentration of MG without any treatment (mg g^−1^), the dye residual concentration (mg g^−1^) in the liquid phase at time *t* after photocatalysis under UV irradiation, and the PFO photocatalytic degradation rate constant (min^−1^), which can be calculated from the slope of the ln(*C*_0_/*C*_tIII_) *versus t* plot.

## 3 Results and discussion

### 3.1 X-ray analysis of the Fe_3_O_4_ NPs samples

**Fig. 2:**
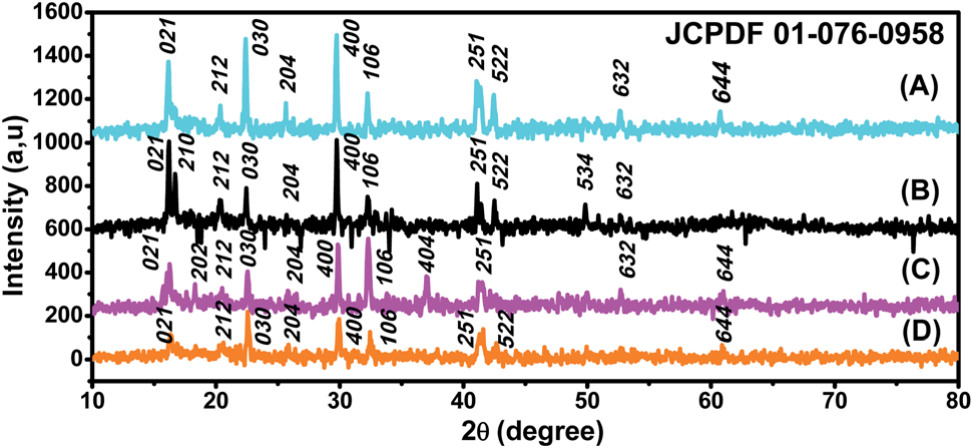
XRD patterns of (A) ROS-Fe_3_O_4_, (B) ARM-Fe_3_O_4_, (C) MAT-Fe_3_O_4_, and (D) JUN-Fe_3_O_4_ NPs. JCPDF file 01-076-0958.

X-ray patterns of all the synthesised samples are presented in Fig. 2. It is found that all synthesized samples have crystalline structures. The X-ray diffraction pattern (A) in Fig. 2 exhibits Bragg reflection peaks at around 2*θ*° = 16.20°, 20.30°, 22.39°, 25.60°, 29.72°, 32.30°, 41.05°, 41.39°, 42.48°, and 52.69°. All Bragg peaks are in agreement with those of orthorhombic Fe_3_O_4_ powder and correspond to the Miller indices 021, 212, 030, 400, 106, 001, 250, 251, 522, and 644, respectively (JCPDF file 01-076-0958).

The X-ray diffraction pattern (B) in Fig. 2 exhibits Bragg reflection peaks at around 2*θ*° = 16.20°, 16.70°, 20.39°, 22.42°, 29.75°, 30.80°, 32.30°, 41.10°, 42.53°, 49.82°, and 52.72°. All Bragg peaks are in agreement with those of orthorhombic Fe_3_O_4_ powder and correspond to the Miller indices 021, 210, 212, 030, 400, 041, 106, 251, 522, 534, and 644, respectively (JCPDF file 01-076-0958).

The X-ray diffraction pattern (C) in Fig. 2 exhibits Bragg reflection peaks at around 2*θ*° = 16.20°, 18.31°, 22.56°, 26.04°, 32.28°, 37.11°, 41.59°, 49.98°, and 52.69°. All Bragg peaks are in agreement with those of orthorhombic Fe_3_O_4_ powder and corresponding to Miller indices 021, 202, 030, 400, 106, 404, 251, 534, and 644, respectively (JCPDF file 01-076-0958).

The X-ray diffraction pattern (D) in Fig. 2 exhibits Bragg reflection peaks at around 2*θ*° = 16.35°, 20.58°, 22.60°, 25.77°, 29.94°, 32.47°, 41.59°, 42.69°, 49.98°, and 52.69°. All Bragg peaks are in agreement with those of orthorhombic Fe_3_O_4_ powder and corresponding to the Miller indices 021, 212, 030, 400, 001, 106, 251, 522, 534, and 644, respectively (JCPDF file 01-076-0958).

The average diameters of the different Fe_3_O_4_ samples, presented in Table 1, were calculated from the XRD patterns using Scherrer’s equation:^53^22
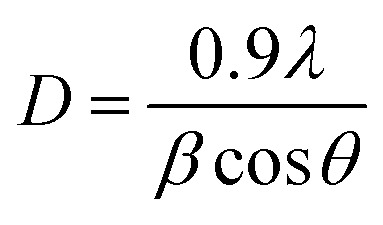
where *D*, *β*, *λ*, and *θ* are the crystallite size, the full width at half-maximum (FWHM) of the most intense diffraction peak, the X-ray wavelength (1.54056 Å), and the Bragg angle, respectively.

**Table 1:** Calculated average diameter of ARM-Fe_3_O_4_, ROS-Fe_3_O_4_, MAT-Fe_3_O_4_ and JUN-Fe_3_O_4_ NPs

Sample	Average diameter (nm)
ARM-Fe_3_O_4_	41.49
ROS-Fe_3_O_4_	39.89
MAT-Fe_3_O_4_	33.13
JUN-Fe_3_O_4_	29.27

### 3.2 FTIR-ATR spectroscopy analysis

**Fig. 3:**
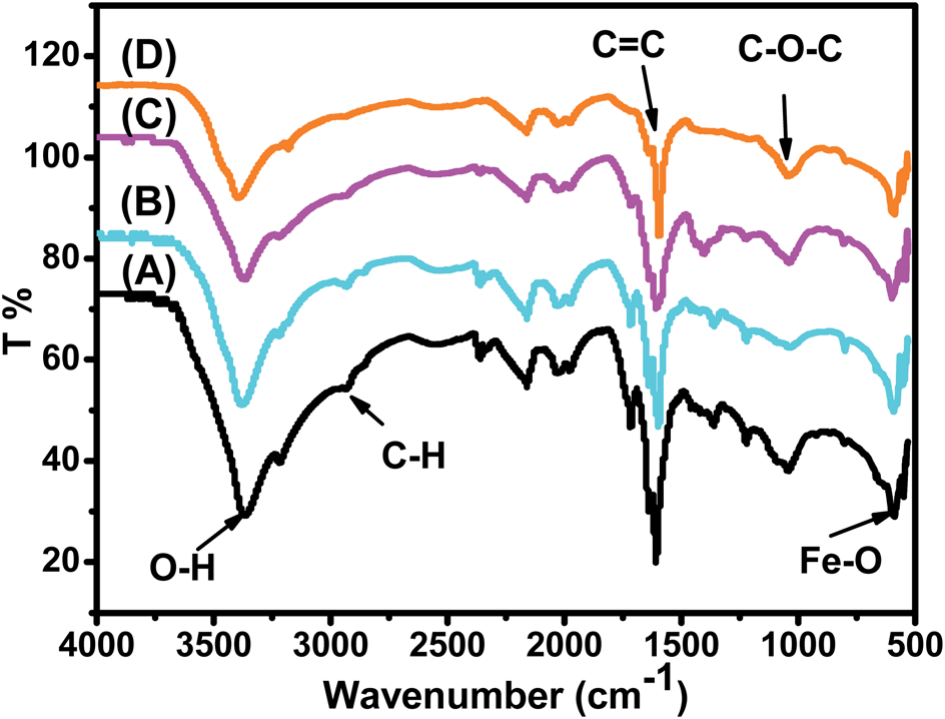
IR spectra of synthesized (A) ARM-Fe_3_O_4_, (B) ROS-Fe_3_O_4_, (C) MAT-Fe_3_O_4_, and (D) JUN-Fe_3_O_4_ NP powders.

The FTIR spectra of the synthesized Fe_3_O_4_ NPs powders recorded between 4000 and 500 cm^−1^ are presented in Fig. 3. Fig. 3 shows that all IR spectra (A, B, C, and D) exhibit peaks in different ranges, as summarized in Table 2. The peaks at 3223.41–3266.69 cm^−1^ correspond to O–H stretching vibrations, whereas the peaks at 2930.18–2932.06 cm^−1^ correspond to C–H vibrations. The peaks at 1590.07–1594.63 cm^−1^ correspond to C=C stretching in aromatic rings and the anti-symmetric stretching of the carboxylate group (COO^−^), whereas peaks at 1033.45–1044.36 cm^−1^ are assigned to the C–O–C of the phenolic groups.^22^ The peak at around 592 cm^−1^ corresponds to the Fe–O stretching band of Fe_3_O_4_ NPs.^54^

**Table 2:** FTIR vibrations of Fe_3_O_4_ functional groups

Sample	O–H (cm^−1^)	C–H (cm^−1^)	CC (cm^−1^)	C–O–C (cm^−1^)	Fe–O (cm^−1^)
ARM-Fe_3_O_4_	3266.69	2932.06	1590.07	1036.36	592.64
ROS-Fe_3_O_4_	3249.77	2930.18	1590.83	1038.75	591.83
MAT-Fe_3_O_4_	3235.57	2929.75	1591.21	1039.54	592.46
JUN-Fe_3_O_4_	3223.41	2928.82	1594.63	1039.45	592.69

Fig. 3 shows that the peaks of hydroxyl groups appear with remarkably different areas. The hydroxyl group peak area appears to be the broadest on the ARM-Fe_3_O_4_ surface, next on ROS-Fe_3_O_4_, then on MAT-Fe_3_O_4_, and finally on JUN-Fe_3_O_4_. This reveals that the density of functional OH groups is higher on the ARM-Fe_3_O_4_ surface, next on ROS-Fe_3_O_4_, then on MAT-Fe_3_O_4_, and finally on JUN-Fe_3_O_4_.

### 3.3 UV-Vis spectroscopy analysis

The optical absorbance spectra of all Fe_3_O_4_ samples were measured in the wavelength range of 200–900 nm. The band gap energies of the Fe_3_O_4_ samples were then deduced from those spectra. The band gap (*E*_g_) and the optical absorption coefficient (*α*) of a semiconductor are related through the known following equation:^55^

**Fig. 4:**
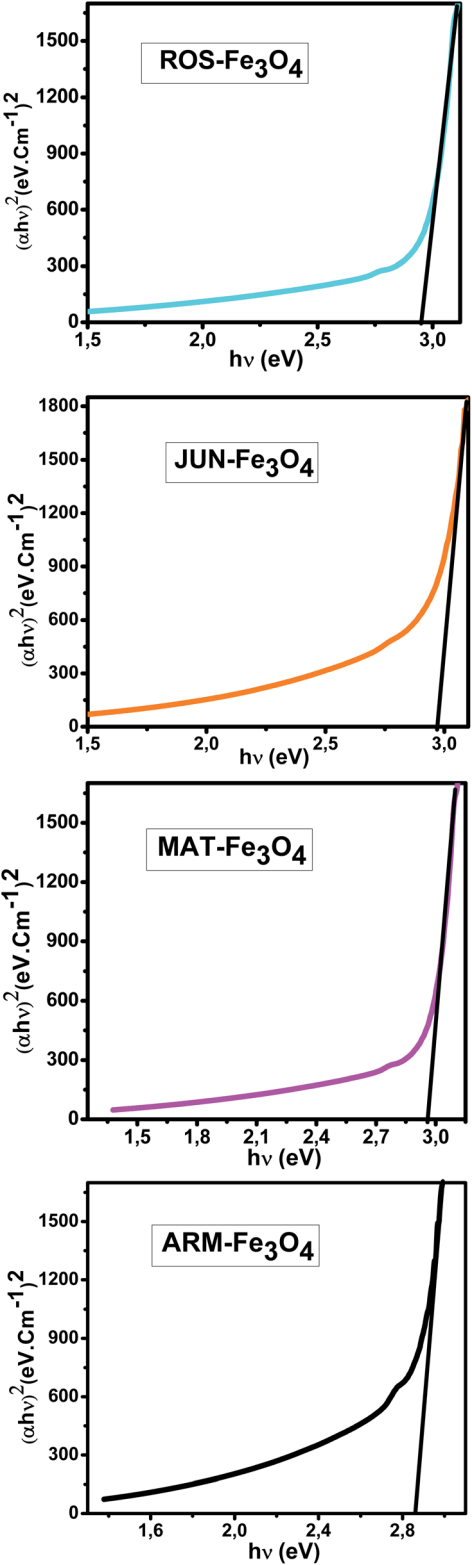
Four plots of (*αhν*)^2^*versus* (*αhν*) for the direct transition of the synthesized Fe_3_O_4_ NP samples sonicated in acetone for 15 min.

**Fig. 5:**
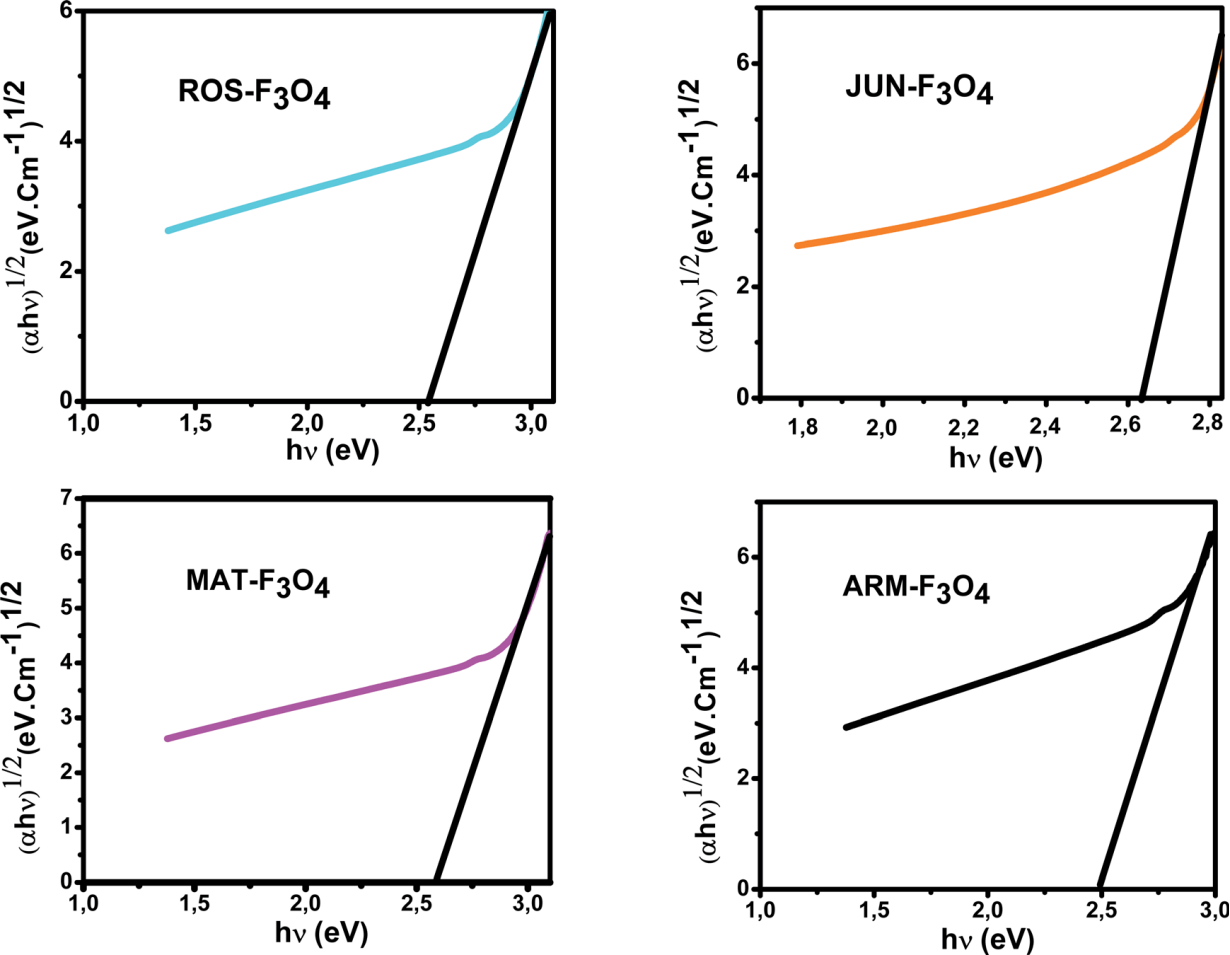
Four plots of (*αhν*)^1/2^*versus* (*αhν*) for the indirect transition of the synthesized Fe_3_O_4_ NP samples sonicated in acetone for 15 min.

23 *αhν* = *A*(*hν*−*E*_g_)^*n*^

where *α* is the linear absorption coefficient of the material, *hν* is the photon energy, *A* is a proportionality constant, and the exponent *n* depends on the nature of electronic transition; it is equal to 1/2 for direct allowed transition and 2 for indirect allowed transition. The *E*_g_ of the direct transition of all samples were obtained from plotting (*αhν*)^2^ as a function of *αhν* by the extrapolation of the linear portion of the curve (Fig. 4). However, the *E*_g_ of the indirect transition of all samples were obtained from plotting (*αhν*)^1/2^ as a function of *αhν* by the extrapolation of the linear portion of the curve (Fig. 5).

The estimated indirect band gap energies of the ARM-Fe_3_O_4_, ROS-Fe_3_O_4_, MAT-Fe_3_O_4_ and JUN-Fe_3_O_4_ samples were found to be 2.51, 2.55, 2.60 and 2.64 eV, respectively, which are higher than the reported reference value.^56^ It was previously found that the indirect gap energy of Fe_3_O_4_ equals *E*_g_ = 1.92 eV. The estimated direct band gap energies of the JUN-Fe_3_O_4_, MAT-Fe_3_O_4_, ROS-Fe_3_O_4_ and ARM-Fe_3_O_4_ samples were found to be 2.97, 2.95, 2.94 and 2.87 eV, respectively, which are close to that found by El Ghandoor *et al.*^56^ They found that the direct gap energy for Fe_3_O_4_ equals *E*_g_ = 2.87 eV. It is clear that the direct gap energy is closer to the theoretical value than the indirect gap energy. The values of all direct band gap energies of the magnetite NP samples classify them as semiconductors. The energy band gaps of semiconductors are between 0 and 3 eV.^57^

### 3.4 SEM images of the greenly synthesised Fe_3_O_4_ NP samples

**Fig. 6:**
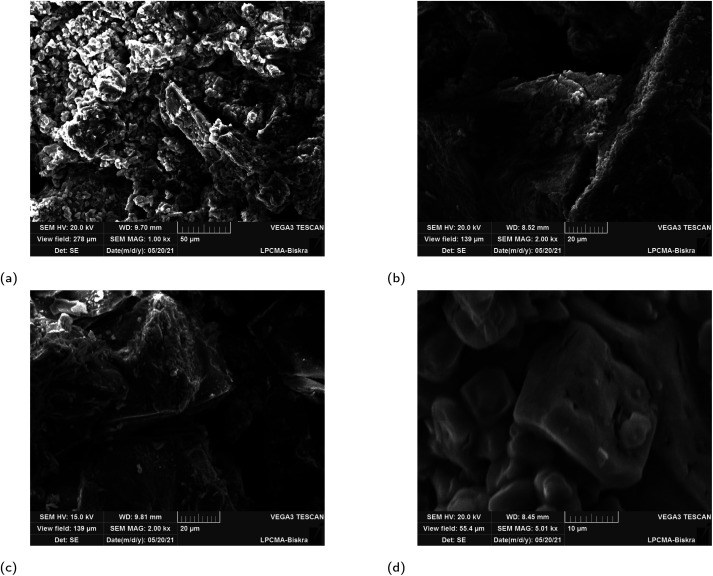
SEM images of greenly synthesized JUN-Fe_3_O_4_, MAT-Fe_3_O_4_, ROS-Fe_3_O_4_ and ARM-Fe_3_O_4_ NPs.

SEM images of the synthesized iron oxide NP samples are presented in Fig. 6. It is clearly shown that the structures of all four magnetite NPs depend on the plant extract. Different irregular shapes are observed in all samples as rock shapes. For ROS-Fe_3_O_4_ NPs, it is clear that a few agglomerations, which appear like rocks, are present, as shown in Fig. 6a. Whereas for the JUN-Fe_3_O_4_ NPs, mountain-like structures with bigger rocks are present, as shown in Fig. 6b. However, for MAT-Fe_3_O_4_, a decrease in the dimension of the mountain-like structures, with more adherence to its structure, is observed (Fig. 6c). Finally, the ARM-Fe_3_O_4_ SEM image contains some big structured single bipyramid crystals, as shown in Fig. 6d.

### 3.5 The analysis of MG adsorption kinetics and thermodynamics

#### 3.5.1 MG adsorption equilibrium in preferential MG adsorption

**Fig. 7:**
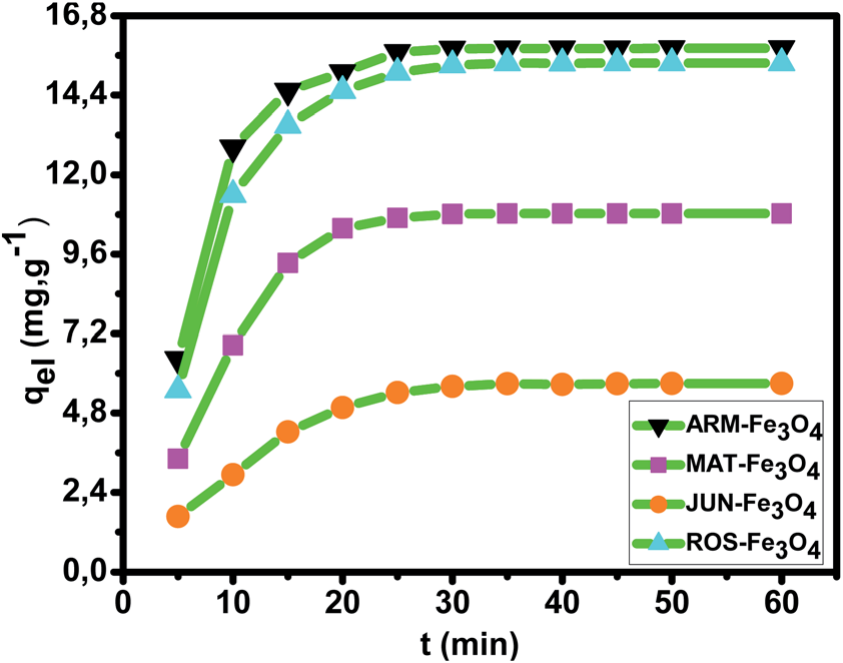
Plots of the MG adsorption capacity (*q*_eI_) *versus* time in the first process of MG adsorption in ambient dark conditions.

In all adsorption experiments, the steady-state is reached within 30 minutes, as depicted in Fig. 7. This represents the very fast adsorption kinetics of MG on all four magnetite NP surfaces.

#### 3.5.2 Pseudo-first-order and pseudo-second-order kinetics in preferential MG adsorption

**Fig. 8:**
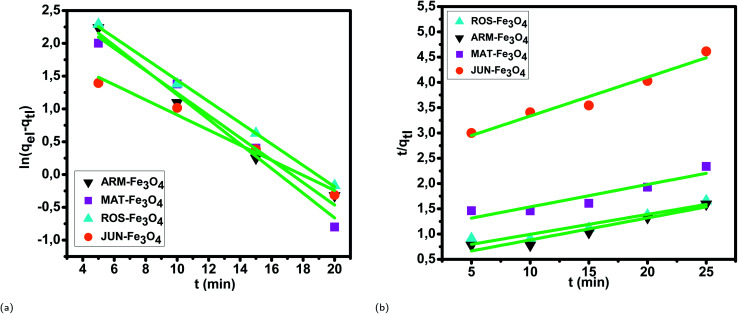
(a) Plots of ln(*q*_eI_ − *q*_tI_) *versus* time and (b) plots of *t*/*q*_tI_*versus* time for MG adsorption on the Fe_3_O_4_ NP samples in the first process of adsorption.

The results of the pseudo-first-order kinetics analysis for preferential MG adsorption on all four magnetite NP surfaces (Table 3 and Fig. 8a) indicate good linearity and a good fit of the experimental data to this model compared to the pseudo-second-order model, which indicated poor linearity and a poor fit of the experimental data (Table 3 and Fig. 8b). The *q*_eI,cal_ (equilibrium adsorption capacity), computed from the pseudo-first-order kinetics plots, are also in very close agreement with the empirical *q*_eI,exp_, contrary to the *q*_eI,cal_ calculated from the pseudo-second-order plots (see Table 3). This indicates the best compliance of MG adsorption on all four magnetite NP surfaces with pseudo-first-order kinetics.

**Table 3:** Adsorption kinetics parameters for MG adsorption on Fe_3_O_4_ NP surfaces in ambient dark conditions

Sample	*q*_eI,exp_ (mg g^−1^)	*q*_eI,cal_ (mg g^−1^)	*K*_1_·10^−3^ (mn^−1^)	*R* ^2^	*q*_eI,cal_ (mg g^−1^)	*K*_2_·10^−3^ (g mg^−1^ mn^−1^)	*R* ^2^
ARM-Fe_3_O_4_	15.81	18.92	5.76	0.9783	22.99	3.78	0.9387
ROS-Fe_3_O_4_	15.37	21.33	9.39	0.9984	25.32	2.60	0.9220
MAT-Fe_3_O_4_	10.83	19.88	8.86	0.9902	22.32	1.85	0.9028
JUN-Fe_3_O_4_	05.69	07.84	8.53	0.9839	13.00	2.31	0.9607

#### 3.5.3 Intra-particle diffusion kinetics in preferential MG adsorption

**Fig. 9:**
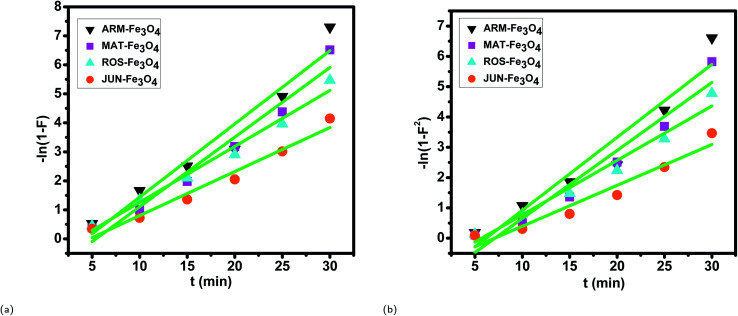
Boyd plots of MG adsorption on Fe_3_O_4_ surfaces in the first process of adsorption: (a) plots of −ln(1 − *F*) *versus* time and (b) plots of −ln(1 − *F*^2^) *versus* time.

The linearity tests of Boyd plots, −ln(1 − *F*) and −ln(1 − *F*^2^) *versus* time, are presented in Fig. 9a and b. They show that the kinetic data correlate well with the homogeneous particle diffusion model, as confirmed by the high *R*^2^ values. The results of linear regression analysis for eqn (9) and (11) are presented in Table 4. It was found that the film diffusion coefficients *D*_f_ were in the order of 10^−11^ m^2^ s^−1^, while the intra-particle diffusion coefficients *D*_p_ were found to be in the order of 10^−19^ m^2^ s^−1^. It is known that the adsorption mechanism is controlled by film diffusion at *D*_f_ ranging from 10^−10^ to 10^−12^ m^2^ s^−1^, while intra-particle diffusion is the rate-limiting step at *D*_p_ in the range of 10^−15^ to 10^−18^ m^2^ s^−1^.^58^ The results found indeed indicate that film diffusion is the step that controls the adsorption mechanism of MG on Fe_3_O_4_ surfaces, which is in agreement with the pseudo-first-order kinetic model.

**Table 4:** Calculated homogeneous particle diffusion parameters in the first process of MG adsorption on plant-Fe_3_O_4_ samples

PLANT-Fe_3_O_4_	*r*_0_·10^−9^ (m)	*k*_p_·10^−3^ (1/s)	*R* ^2^	*D*_p_·10^−19^ (m^2^ s^−1^)	*k*_f_·10^−3^ (1/s)	*R* ^2^	*C*_rI_·10^−3^ (mg g^−1^)	*C*_aI_·10^−3^ (mg g^−1^)	*D*_f_·10^−11^ (m^2^ s^−1^)
ARM	41.94	2.68	0.9411	4.77	5.61	0.9594	5.17	5.93	6.84
ROS	39.89	2.00	0.9736	3.22	4.29	0.9826	5.34	5.76	5.28
MAT	33.13	2.67	0.9645	2.97	5.32	0.9599	7.11	3.99	10.49
JUN	29.27	1.50	0.9544	1.30	3.38	0.9701	8.96	2.14	13.81

#### 3.5.4 Activation thermodynamic parameters of MG adsorption under the thermocatalysis process

**Fig. 10:**
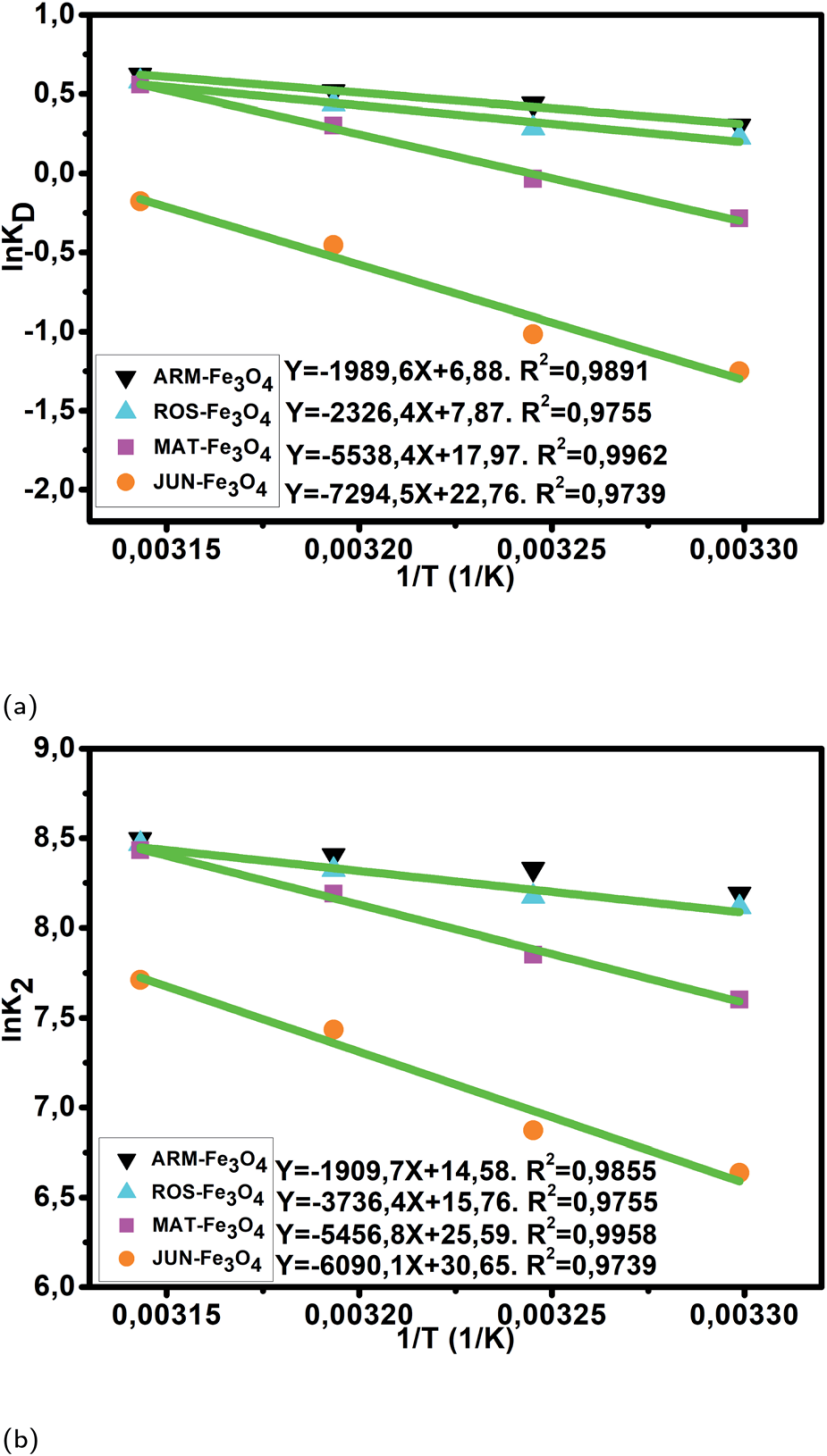
(a) Plots of ln *K*_D_*versus* 1/*T* of MG adsorption on Fe_3_O_4_ surfaces, (b) plots of lnv*K*_2_*versus* 1/*T* of MG adsorption on Fe_3_O_4_ NP surfaces.

The calculated activated enthalpy (Δ*H*^0^), entropy (Δ*S*^0^), and free energy (Δ*G*^0^) are listed in Table 5. Δ*H*^0^ and Δ*S*^0^ are respectively calculated from the slopes and intercepts of the Arrhenius linear plots of ln *k*_D_*versus* 1/*T* (Fig. 10a). The activated enthalpies in all four MG/Fe_3_O_4_ systems are positive, which indicates the endothermic nature of the adsorption processes and possible strong bonding between MG molecules and functional hydroxyl groups on Fe_3_O_4_ surfaces. The found activated enthalpy of the MG/JUN-Fe_3_O_4_ system (14.49 kcal mol^−1^) is the highest one, and that of the MG/ARM-Fe_3_O_4_ system (4.59 kcal mol^−1^) is the lowest one. This indicates that the bonding between MG molecules and hydroxyl groups on the JUN-Fe_3_O_4_ NP surface is the strongest, then on MAT-Fe_3_O_4_, next on ROS-Fe_3_O_4_, and finally on the ARM-Fe_3_O_4_ NP surface.

**Table 5:** Calculated thermodynamic parameters for MG adsorption on Fe_3_O_4_ NP surfaces in the second process of adsorption in dark conditions

Sample	*T* (K)	ln *K*_D_	ln *K*_2_	*E*_a_ (kcal mol^−1^)	Δ*H*^0^ (kcal mol^−1^)	Δ*S*^0^ (cal mol^−1^ K^−1^)	Δ*G*^0^ (kcal mol^−1^)
MG/ARM-Fe_3_O_4_	303.15	0.30	8.19	3.79	3.95	13.48	−0.18
	308.15	0.44	8.33				−0.27
	313.15	0.52	8.41				−0.32
	318.15	0.62	8.49				−0.39
MG/ROS-Fe_3_O_4_	303.15	0.23	8.17	7.42	4.62	15.64	−0.14
	308.15	0.29	8.31				−0.17
	313.15	0.44	8.61				−0.27
	318.15	0.58	8.72				−0.37
MG/MAT-Fe_3_O_4_	303.15	−0.28	7.60	10.84	11.00	35.70	0.17
	308.15	−0.04	7.85				0.02
	313.15	0.30	8.19				−0.19
	318.15	0.56	8.43				−0.35
MG/JUN-Fe_3_O_4_	303.15	−1.25	6.64	12.10	14.49	45.23	0.75
	308.15	−1.01	6.87				0.62
	313.15	−0.45	7.44				0.28
	318.15	−0.18	7.71				0.11

The activated entropies in all four MG/Fe_3_O_4_ systems are positive, which reveals the affinity of the Fe_3_O_4_ surfaces for MG molecules. The increasing randomness at the MG/Fe_3_O_4_ solution interface indicates that a highly significant change in the surface active hydroxyl group number occurred in the internal structure of the Fe_3_O_4_ surfaces. However, the activated entropy of the MG/JUN-Fe_3_O_4_ system is the highest one (45.23 cal mol^−1^ K^−1^), and that of MG/ARM-Fe_3_O_4_ (15.58 cal mol^−1^ K^−1^) is the lowest one. This indicates that the changes occurring in the structure of the JUN-Fe_3_O_4_ NPs’ surface are the largest ones, then of MAT-Fe_3_O_4_, next of ROS-Fe_3_O_4_, and finally of the ARM-Fe_3_O_4_ NP surface.^59,60^

The activated free energies of the MG/ARM-Fe_3_O_4_ (−0.18, −0.24, −0.32, and −0.39 kcal mol^−1^) and MG/ROS-Fe_3_O_4_ (−0.14, −0.17, −0.27, and −0.37 kcal mol^−1^) systems are both negative. However, the activated energies of the MG/ARM-Fe_3_O_4_ system are more negative than those of the MG/ROS-Fe_3_O_4_ system, which indicates the feasibility of the MG adsorption process and its spontaneous nature, with more MG adsorption on ARM-Fe_3_O_4_ than on the ROS-Fe_3_O_4_ surface. In the MG/MAT-Fe_3_O_4_ system, the values of the activated free energies are negative only at 313.15 K and 318.15 K (−0.19 and −0.35 kcal mol^−1^, respectively), while positive values are found at 303.15 K and 308.15 K (0.17 and 0.022 kcal mol^−1^, respectively). This demonstrates the spontaneity of MG adsorption at 313.15 K and 318.15 K. The activated free energies of the MG/JUN-Fe_3_O_4_ system (0.75, 0.62, 0.28, and 0.11 kcal mol^−1^) are positive, revealing that the activated MG/Fe_3_O_4_ complexes are in an excited form in the transition state.^59^

As presented in Table 5, the found activation energies (*E*_a_) for MG adsorption on the ARM-Fe_3_O_4_, ROS-Fe_3_O_4_, MAT-Fe_3_O_4_ and JUN-Fe_3_O_4_ surfaces are, respectively, 4.43, 7.42, 10.84, and 12.10 kcal mol^−1^. *E*_a_ is calculated from the slopes of the Arrhenius linear plots of ln *k*_2_*versus* 1/*T* (Fig. 10b). The found low *E*_a_ suggests that MG adsorption on Fe_3_O_4_ proceeds with a low energy barrier and can be achieved at relatively low temperatures. As it is known that the activation energy *E*_a_ of physical adsorption ranges from 1.2 to 12 kcal mol^−1^, and from 14.3 to 191 kcal mol^−1^ for chemical adsorption,^61^ the adsorption processes of MG on all four Fe_3_O_4_ samples are therefore physical in nature.

#### 3.5.5 Pseudo-first-order kinetic analysis of MG adsorption under the photocatalysis process

**Fig. 11:**
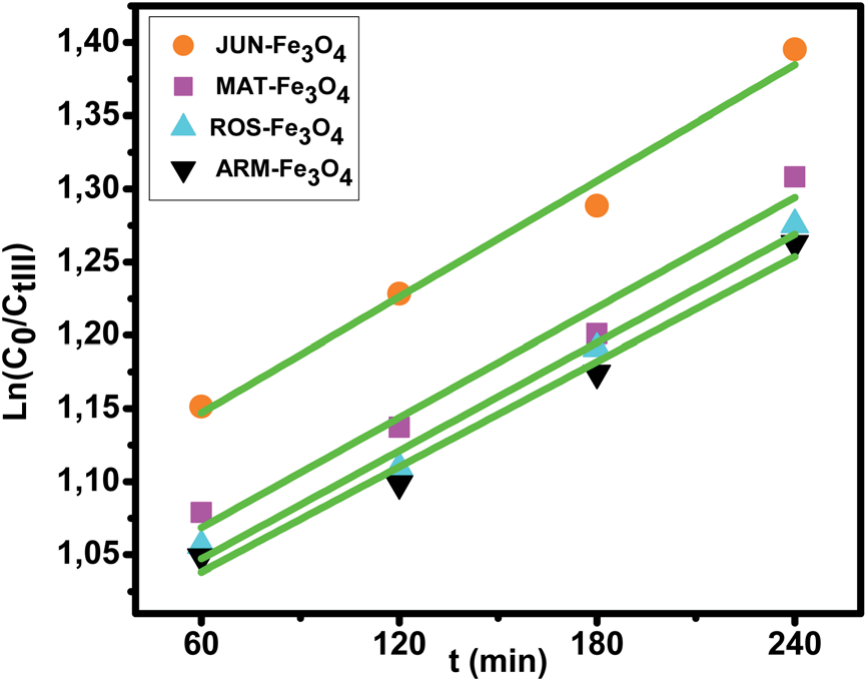
Kinetic plots of ln*C*_0_/*C*_tIII_*versus* time of MG photodegradation on the Fe_3_O_4_ NP samples.

The results of the pseudo-first-order kinetic analysis of MG adsorption on the four magnetite NP surfaces (Fig. 11) indicate a good linearity of the plots of ln(*C*_0_/*C*_tIII_) *versus* time of UV irradiation, as judged from the high correlation coefficients (*R*^2^ > 0.98), which indicate that the rate of MG degradation catalyzed by the Fe_3_O_4_ NP samples is able to be fitted by a pseudo-first-order model. The corresponding photodegradation rates (*k*_pd_) of MG by JUN-Fe_3_O_4_, MAT-Fe_3_O_4_, ROS-Fe_3_O_4_, and ARM-Fe_3_O_4_ are 0.00132 min^−1^, 0.00125 min^−1^, 0.00123 min^−1^, and 0.00120 min^−1^, respectively.

### 3.6 Preferential and enhanced MG adsorption on magnetite surfaces

#### 3.6.1 Preferential MG adsorption

Table 6 shows that, in the first process of MG adsorption in ambient dark conditions, the adsorption capacity and yield of MG differ depending on the Fe_3_O_4_ NP sample. The MG adsorption capacity (adsorption capacity is denoted *q*_eI_) and yield (denoted *R*_I_%) achieved were 15.81 mg g^−1^ and 53.42% on ARM-Fe_3_O_4_ and 15.37 mg g^−1^ and 51.90% on ROS-Fe_3_O_4_, respectively. Whereas only 10.83 mg g^−1^ and 35.91% on MAT-Fe_3_O_4_ and 19.25% and 5.70 mg g^−1^ on JUN-Fe_3_O_4_ surfaces was achieved. So, MG molecules are highly adsorbed on ARM-Fe_3_O_4_, next on ROS-Fe_3_O_4_, then on MAT-Fe_3_O_4_, and finally on the JUN-Fe_3_O_4_ NP surfaces. As all experimental conditions were kept the same for all adsorption experiments on all four magnetite samples, only the magnetite surface’s functionality is responsible for the preferential adsorption of MG on the magnetite NP surfaces.

**Table 6:** Achieved MG adsorption capacity and yield on ARM-Fe_3_O_4_, ROS-Fe_3_O_4_, MAT-Fe_3_O_4_ and JUN-Fe_3_O_4_ NPs in the first process of MG adsorption in ambient dark conditions

Adsorbent	*q*_eI_ (mg g^−1^)	MG *R*_I_%
ARM-Fe_3_O_4_	15.81	53,42
ROS-Fe_3_O_4_	15.37	51.90
MAT-Fe_3_O_4_	10.83	35.91
JUN-Fe_3_O_4_	05.70	19.25

It is known that complexation and electrostatic interactions play important roles in determining the efficiency of adsorption.^6^ When Fe_3_O_4_ is immersed in the aqueous acidic solution, it develops its surface charge *via* the protonation and deprotonation of ≡Fe–OH active sites on its surface according to the following equation:^62^

24 ≡Fe–OH_2_^+^ ↔ ≡FeOH^0^ + H_sol_^+^ (*p*K_a1_ = 5.1)

where ≡Fe–OH_2_^+^ and ≡Fe–OH^0^ are, respectively, the protonated positively charged acid site of the surface with two dissociable H^+^, and the neutral acid site of the surface with one dissociable H^+^. *p*K_a1_ = 5.1 is the intrinsic acidity constant determined by Davis *et al.*^62^ for Fe_3_O_4_. The binding of MG cations with functional groups, such as OH, from the magnetite surface can be expressed as follows:

25 2≡Fe–OH + dye^2+^ ↔ ≡(Fe–OH)_2_–dye^2+^

where ≡(Fe–OH)_2_–dye^2+^ is a binuclear bonding complex due to hydrogen bonding between MG cations and the surface hydroxyl groups on magnetite NP surfaces.

For the four magnetite NPs, the data provided by FTIR analysis (see Section 3.2, Fig. 3) show that the density of OH groups on the ARM-Fe_3_O_4_ surface is the highest one, next to ROS-Fe_3_O_4_, then MAT-Fe_3_O_4_, and finally JUN-Fe_3_O_4_. As these hydroxyl groups behave as active sites on the Fe_3_O_4_ surfaces, the results found show that MG adsorption yield is more increased on magnetite NP samples that have more OH groups, *i.e.* more ≡Fe–OH active sites.

#### 3.6.2 Enhancement of MG adsorption by the thermocatalysis process

To assess the MG adsorption enhancement by thermocatalysis, the thermocatalytic experiments were conducted on MG/Fe_3_O_4_ residual solutions after MG adsorption in the first process, so as to give the overall adsorption yield and capacity after the enhancement, and it also allows the comparison between adsorption yields and capacities before and after thermocatalysis.

**Fig. 12:**
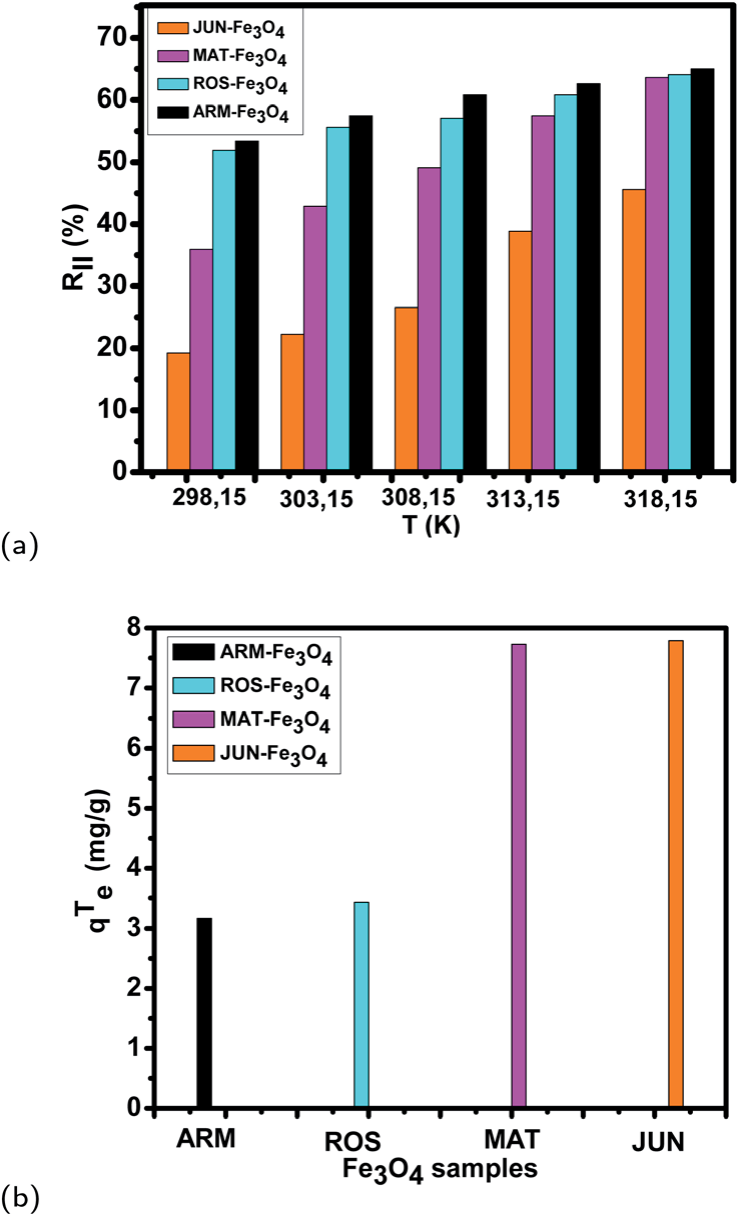
(a) MG adsorption yield on Fe_3_O_4_ surfaces under thermocatalysis in a temperature range from 303.15 K to 318.15 K for 20 minutes in dark conditions. (b) The enhancement in the adsorption capacity *q*^T^_e_ by thermocatalysis in dark conditions at 318.15 K for 20 minutes of MG adsorption on the four Fe_3_O_4_ surfaces.

The thermocatalysis effect on MG adsorption on all four magnetite samples was evaluated by assessing the efficiency of the degradation of MG by thermocatalysis in dark conditions in a temperature range from 303.15 K to 318.15 for 20 minutes. Fig. 12a and Table 7 present the comparison of the MG adsorption yield and capacity of the four Fe_3_O_4_ surfaces in the first process of MG adsorption and in the second process in dark conditions under thermocatalysis. The data show that the MG adsorption yield and capacity increase with the increase of temperature in all adsorption experiments, which confirms the endothermic nature of the adsorption processes, as discussed in Section 3.5.4.

**Table 7:** The enhancement of the MG adsorption yield and capacity on the Fe_3_O_4_ NP surfaces by thermocatalysis

Sample	298.15 K		303.15 K		308.15 K		313.15 K		318.15 K	
	*q*_eI_ (mg g^−1^)	*R*_I_%	*q*_eII_ (mg g^−1^)	*R*_II_%	*q*_eII_(mg g^−1^)	*R*_II_%	*q*_eII_(mg g^−1^)	*R*_II_%	*q*_eII_ (mg g^−1^)	*R*_II_%
MG/ARM-Fe_3_O_4_	15.81	53.42	17.01	57.48	18.00	60.81	18.53	62.61	18.98	65.01
MG/ROS-Fe_3_O_4_	15.37	51.90	16.32	55.64	16.75	57.09	17.81	60.83	18.80	64.09
MG/MAT-Fe_3_O_4_	10.83	35.91	12.69	42.88	14.53	49.10	17.01	57.48	18.56	63.60
MG/JUN-Fe_3_O_4_	05.70	19.25	06.59	22.25	07.87	26.58	11.49	38.83	13.49	45.59

Yields and adsorption capacities are increased as follows (the yield after thermocatalysis is denoted as *R*_II_% and the adsorption capacity as *q*_eII_):

• On ARM-Fe_3_O_4_, the yield increased from *R*_I_% = 53.42% to *R*_II_% = 65.01%, and the adsorption capacity increased from *q*_eI_ = 15.81 to *q*_eII_ = 18.98 mg g^−1^.

• On ROS-Fe_3_O_4_, the yield increased from *R*_I_% = 51.90% to *R*_II_% = 64.09%, and the adsorption capacity increased from *q*_eI_ = 15.37 to *q*_eII_ = 18.80 mg g^−1^.

• On MAT-Fe_3_O_4_, the yield increased from *R*_I_% = 35.91% to *R*_II_% = 63.60%, and the adsorption capacity increased from *q*_eI_% = 10.83 to *q*_eII_% = 18.56 mg g^−1^.

• On JUN-Fe_3_O_4_, the yield increased from *R*_I_% = 19.25% to *R*_II_% = 45.59%, and the adsorption capacity increased from *q*_eI_ = 5.70 to *q*_eII_ = 13.49 mg g^−1^.

As all experiment conditions were kept the same for all adsorption experiments, only the surface properties are responsible for the adsorption enhancement.

The tendency of adsorption capacities and yields on the four magnetite NP surfaces is the same in the first process of MG adsorption and in the second process of MG adsorption under thermocatalysis. In the first process of MG adsorption, the highest adsorption capacity was on ARM-Fe_3_O_4_ NPs, then on ROS-Fe_3_O_4_ NPs, next on MAT-Fe_3_O_4_ NPs, and finally on JUN-Fe_3_O_4_ NPs. After the exposure of MG/Fe_3_O_4_ systems to heat at 303.15 K for 20 minutes, the order of the adsorption capacities was the same, where the highest adsorption capacity was on ARM-Fe_3_O_4_, then on ROS-Fe_3_O_4_ NPs, next on MAT-Fe_3_O_4_ NPs, and finally on JUN-Fe_3_O_4_ NP surfaces. When further exposing the MG/Fe_3_O_4_ systems to heat at different temperatures of 308.15, 313.15, and 318.15 K for 20 minutes, the adsorption capacities of all four magnetite samples still increased in the same order. As shown in Fig. 12b, it is clear that there is a unique difference in the adsorption capacities after thermocatalysis, denoted as *q*^T^_e_ (*q*^T^_e_ = *q*_eII_−*q*_eI_ represents the enhancement in the adsorption capacity by thermocatalysis calculated as the difference between *q*_eI_, the adsorption capacity in the first process of MG adsorption, and *q*_eII_, the overall adsorption capacity after carrying out thermocatalysis at 318.15 K for 20 minutes). Fig. 12b presents *q*^T^_e_ for the four MG/Fe_3_O_4_ systems. These adsorption capacities are useful to elucidate the enhancement in MG adsorption by thermocatalysis. They show that *q*^T^_e_ is the highest on JUN-Fe_3_O_4_ (7.79 mg g^−1^) and the lowest on ARM-Fe_3_O_4_ (3.17 mg g^−1^). This indicates that the thermocatalytic activity of the JUN-Fe_3_O_4_ NPs is the highest and that of ARM-Fe_3_O_4_ is the lowest. As all experimental conditions were kept the same for all adsorption experiments on all four magnetite samples, only the magnetite surfaces’ properties are responsible for the adsorption enhancement by the thermocatalysis process.

From Fig. 12b and Table 7, it can be seen that the increase in *q*^T^_e_ is accompanied by an increase in Δ*S*^0^ in all the MG-Fe_3_O_4_ systems (detailed in Section 3.5.4). This confirms that the increase in *q*^T^_e_ has resulted from the change in the surface structure.^60^ Thus, the maximum changes occurred in the structural surface of the JUN-Fe_3_O_4_ NPs, and the minimum changes occurred in the structural surface of the ARM-Fe_3_O_4_ NPs.

#### 3.6.3 Enhancement of MG adsorption by the photocatalysis process

To assess the MG adsorption enhancement by photocatalysis, the photocatalytic experiments were conducted on MG/Fe_3_O_4_ residual solutions after the thermocatalytic experiments, so as to give the overall adsorption yields and capacities after the enhancement by photocatalysis and allow the comparison between the adsorption yields and capacities before and after carrying out the photocatalysis process. The impact of the photocatalysis process on MG adsorption on all four magnetite samples was evaluated by assessing the efficiency of the degradation of MG under UV irradiation (365 nm) in a time range from 60 to 240 minutes in ambient conditions.

**Fig. 13:**
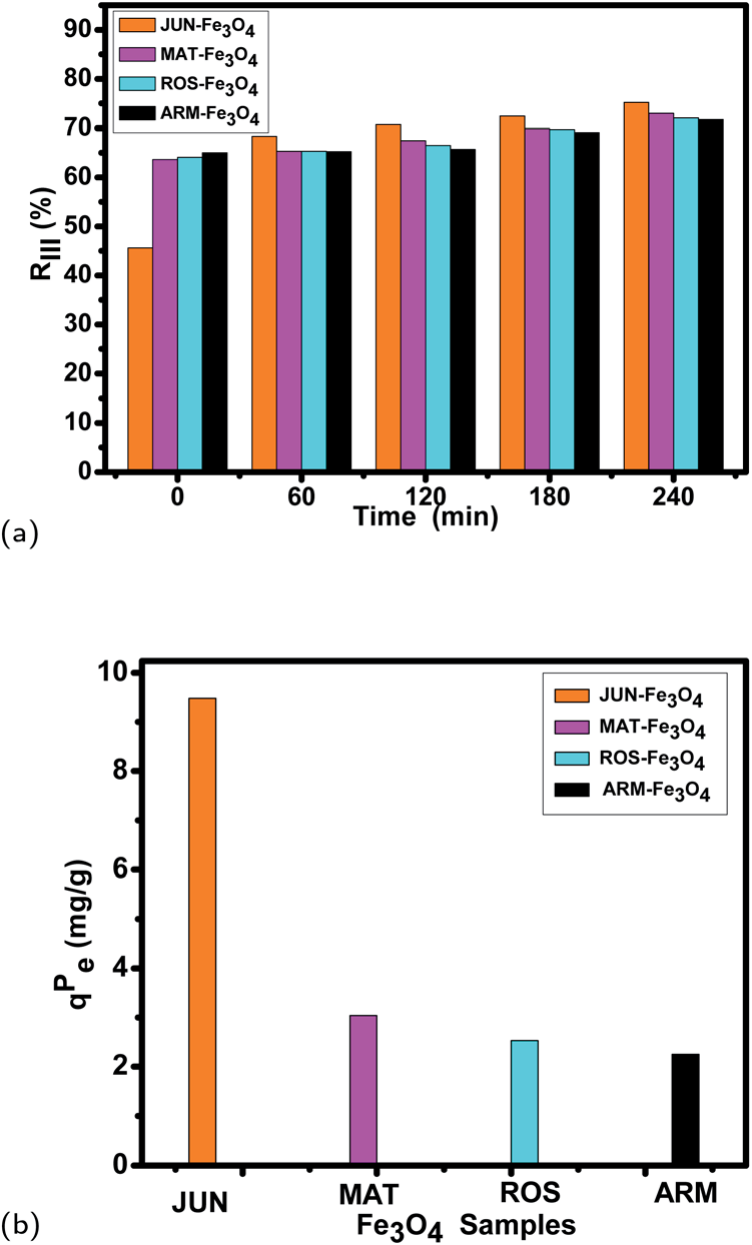
(a) MG adsorption yield on Fe_3_O_4_ NP surfaces under photocatalysis in ambient conditions, (b) the enhancement of MG adsorption *q*^P^_e_ under photocatalysis in ambient conditions for 240 min of MG adsorption on the four Fe_3_O_4_ surfaces.

The variation of the MG adsorption yields, as well as the adsorption capacities under photocatalysis, is illustrated in Table 8 and Fig. 13a. They show that the MG adsorption capacities and yields on the four magnetite surfaces are enhanced by photocatalysis, however with remarkably different differences. Table 8 and Fig. 13a show remarkable differences when comparing the adsorption results on the four magnetite samples before carrying out photocatalysis and after 240 minutes of exposure to UV irradiation in ambient conditions, where the MG adsorption yield and adsorption capacity vary as follows (the yield after carrying out photocatalysis is denoted as *R*_III_% and the adsorption capacity as *q*_eIII_):

• On ARM-Fe_3_O_4_, the yield increased from *R*_II_% = 65.01% to *R*_III_% = 71.71%, and the adsorption capacity increased from *q*_eII_ = 18.98 to *q*_eIII_ = 21.23 mg g^−1^.

• On ROS-Fe_3_O_4_, the yield increased from *R*_II_% = 64.09% to *R*_III_% = 72.07%, and the adsorption capacity increased from *q*_eII_ = 18.80 to *q*_eIII_ = 21.33 mg g^−1^.

• On MAT-Fe_3_O_4_, the yield increased from *R*_II_% = 63.60% to *R*_III_% = 72.97%, and the adsorption capacity increased from *q*_eII_ = 18.56 to *q*_eIII_ = 21.60 mg g^−1^.

• On JUN-Fe_3_O_4_, the yield increased from *R*_II_% = 45.59% to *R*_III_% = 75.23%, and the adsorption capacity increased from *q*_eII_ = 13.49 to *q*_eIII_ = 22.27 mg g^−1^.

**Table 8:** MG adsorption yield and capacity on Fe_3_O_4_ NP surfaces under photocatalysis in ambient conditions

Sample	0 min		60 min		120 min		180 min		240 min	
	*q*_eII_(mg g^−1^)	*R*_II_%	*q*_eIII_ (mg g^−1^)	*R*_III_%	*q*_eIII_ (mg g^−1^)	*R*_III_%	*q*_eIII_ (mg g^−1^)	*R*_III_%	*q*_eIII_ (mg g^−1^)	*R*_III_%
MG/ARM-Fe_3_O_4_	18.98	65.01	19.31	65.23	19.44	65.68	20.45	69.10	21.23	71.71
MG/ROS-Fe_3_O_4_	18.80	64.09	19.33	65.32	19.68	66.49	20.64	69.73	21.33	72.07
MG/MAT-Fe_3_O_4_	18.56	63.60	19.34	65.32	19.97	67.48	20.69	69.91	21.60	72.98
MG/JUN-Fe_3_O_4_	13.49	45.52	20.24	68.38	20.93	70.72	21.44	72.44	22.97	75.23

In the first process of MG adsorption, the highest adsorption capacity was on the ARM-Fe_3_O_4_ NPs, then on ROS-Fe_3_O_4_ NPs, next on MAT-Fe_3_O_4_ NPs, and finally on JUN-Fe_3_O_4_ NPs. Whereas after the exposure of the MG/Fe_3_O_4_ systems to UV irradiation for 60 minutes, the order of the adsorption capacities was inverted, where the highest adsorption capacity was on the JUN-Fe_3_O_4_ NPs, then on MAT-Fe_3_O_4_ NPs, next on ROS-Fe_3_O_4_ NPs, and finally on ARM-Fe_3_O_4_ NPs. When further exposing the MG/Fe_3_O_4_ systems to UV irradiation for 120, 180, and 240 minutes, the adsorption capacities of all four magnetite samples still increased in the same order.

As shown in Fig. 13b, it is evident that there is a clear difference between the adsorption capacities under photocatalysis, denoted as *q*^P^_e_ (*q*^P^_e_ = *q*_eIII_ − *q*_eII_ represents the enhancement in adsorption by photocatalysis, it is calculated from the difference between the overall adsorption capacity *q*_eIII_ in the third process of the adsorption after carrying out photocatalysis for 240 minutes, and the adsorption capacity *q*_eII_ in the second process of MG adsorption after carrying out thermocatalysis at 318.15 K for 20 minutes) for the four MG/Fe_3_O_4_ systems. These *q*^P^_e_ are useful to elucidate the MG adsorption enhancement by photocatalysis. They show that the highest one is that of JUN-Fe_3_O_4_ (9.48 mg g^−1^) and the lowest one is that of ARM-Fe_3_O_4_ (2.27 mg g^−1^). This indicates that the photocatalytic activity of JUN-Fe_3_O_4_ is the highest and that of ARM-Fe_3_O_4_ is the lowest. As all experimental conditions were kept the same for all adsorption experiments on all four magnetite samples, only the magnetite surfaces’ properties are responsible for the adsorption enhancement by the photocatalysis process.

### 3.7 Influence of the mediating plant extract’s acidity on the preferential and enhanced MG adsorption on magnetite surfaces

The results from the analysis of MG adsorption in ambient dark conditions showed that MG was differently adsorbed on the four magnetite surfaces (see Table 6). When comparing the OH group densities on the magnetite surfaces (according to the FTIR spectra analyzed in Section 3.2), it was found that MG adsorption is more preferred on magnetite surfaces that have more OH groups. The adsorption yield was the highest on the ARM-Fe_3_O_4_ surface, next on ROS-Fe_3_O_4_, then on MAT-Fe_3_O_4_, and finally on JUN-Fe_3_O_4_ and their mediating plant extracts have, respectively, acidic pH values of 5.25, 5.05, 4.63, and 3.69. Therefore, one can conclude that the plant extract pH has a clear effect on the OH group density on the magnetite surfaces and, consequently, on the preferential adsorption of MG. Thus, the decrease in the mediating plant extract’s acidity led to the increase in MG adsorption on the greenly synthesized magnetite NPS.

Furthermore, the results found showed that the particle sizes of the magnetite samples vary with the variation of the plant extract mediating their green synthesis. The average grain size of the JUN-Fe_3_O_4_, MAT-Fe_3_O_4_, ROS-Fe_3_O_4_, and ARM-Fe_3_O_4_ NPs, calculated using Scherrer’s equation (eqn (22)), are, respectively, 29.27, 33.13, 39.89 and 41.49 nm, and their mediating extracts’ acidic pH were, respectively, 3.69, 4.63, 5.05, and 5.25. Therefore, the particle size decreases with the increase of the plant extract’s acidity. This result is in agreement with that found by Makarov *et al.*^63^ Moreover, the band gap energies of the JUN-Fe_3_O_4_, MAT-Fe_3_O_4_, ROS-Fe_3_O_4_, and ARM-Fe_3_O_4_ NPs are, respectively, 2.97, 2.95, 2.94, and 2.87. The smaller crystallite size of Fe_3_O_4_ is related to the higher band gap energy value as proof of the quantum size effect. So, the decrease of particle size leads to an increase in the band gap energy. This result is in agreement with that reported by Singh *et al.*^64^ Therefore, one can conclude that the band gap energy increases with the increase of the plant extract’s acidity.

The photo- and thermocatalysis adsorption mechanisms are controlled by the photo- and thermogenerated electron/hole pairs, which exhibit a strong tendency to recombine. The lifetime of the electron/hole pairs influences the photo- and thermocatalytic efficiency.^15,65,66^ The results found showed that the thermo- and photocatalytic activities of the magnetite NPs samples differ according to the mediating plant extract’s acidity. *q*^P^_e_ and *q*^T^_e_ are the highest on JUN-Fe_3_O_4_, next on MAT-Fe_3_O_4_, then on ROS-Fe_3_O_4_, and finally on the ARM-Fe_3_O_4_ NPs. This indicates that the recombination lifetime of the electron/hole pairs was more decreased on the JUN-Fe_3_O_4_ surface, next on MAT-Fe_3_O_4_, then on ROS-Fe_3_O_4_, and finally on the ARM-Fe_3_O_4_ NPs. Seeing that the increase in the direct band gap energy further slows the electron/hole pair recombination,^16^ and the band gap energy increases with the increase of the plant extract’s acidity, thus one can pronounce that the plant extract has an effect on the recombination lifetime of the electron/hole pairs, where the recombination of the electron/hole pairs is further slowed by the increase of the plant extract’s acidity. Thus, the thermo- and photocatalysis enhance the MG adsorption yields and capacities more on magnetite surfaces that are greenly synthesized from more acidic mediating plant extracts. Magnetite NPs greenly synthesized from more acidic mediating plant extracts showed higher thermo- and photocatalytic activities for MG adsorption.

## 4 Conclusion

The preferential and enhanced MG adsorption by thermo- and photocatalysis on four greenly synthesized magnetite surfaces has been studied by coupling three processes. In the first process, MG adsorption on magnetite surfaces was conducted in ambient dark conditions, whereas in the second and third processes, the enhancement by thermo- and photocatalysis were measured in dark conditions and under UV irradiation (365 nm) in ambient conditions, respectively. All four greenly synthesized magnetite samples were characterized by XRD, SEM, ATR-FTIR, and UV-Vis.

The results found showed that:

• The decrease in the plant extract’s acidity leads to the increase of the active site density and, hence, an increase in the MG adsorption yield and capacity.

• The mediating plant extract’s acidity clearly affects the adsorption enhancement by thermo- and photocatalysis through its effect on the band gap energy of the greenly synthesized magnetite and, consequently, on the recombination lifetime of the electron/hole pairs after electron excitation.

• The band gap energy increases with the increase of the plant extract’s acidity, and the recombination speed of the electron/hole pairs is further decreased by the increase of the plant extract’s acidity.

Therefore, the thermo- and photocatalysis processes enhance the MG adsorption yield and capacity more on magnetite surfaces that are greenly synthesized from more acidic mediating plant extracts.

## Author contributions

Kaouthar Ahmouda: writing – original draft, resources, investigation, visualization, and methodology. Boubakeur Benhaoua: supervision and visualization.

## Conflicts of interest

There are no conflicts to declare.

## Supplementary Material
